# Dual infection with Ehrlichia chaffeensis and a spotted fever group rickettsia: a case report.

**DOI:** 10.3201/eid0404.980430

**Published:** 1998

**Authors:** A J Sulzer


Among the 54 reporting systems for which further information was obtained, clinical diagnoses (in some countries laboratory confirmed) are reported through the hierarchical chain, normally by mail or facsimile, but in two countries by electronic links. Almost all military reporting systems are parallel to civilian systems. Thirty-four (63%) of 54 systems feed into the civilian system, with a built-in mechanism to avoid duplicate reporting; 16 (30%) systems feeding into the civilian system have no such mechanism in place; and four have no link with the civilian system.

The third survey addressed vaccination policies. Among 52 countries that replied, 47 (90%) have a compulsory military vaccination schedule: 45 (87%) for tetanus, 30 (58%) for diphtheria, 23 (44%) for typhoid, 16 (31%) for bacillus Calmette-Guérin and polio, 12 (23%) for meningococcal meningitis, and 10 (19%) for measles, mumps, and rubella.

These surveys show that military populations are protected against many infectious diseases and that a wealth of information is obtained by military laboratories and health-care facilities on populations at high risk for infectious diseases. While most of the information collected from the health-care facilities is reported through civilian systems as well, incorporating the military network of laboratories into the WHO global surveillance network could ensure broader coverage.

**Raffaele D'Amelio*† and David L. Heymann†**
*Ministero della Difesa, Direzione Generale Sanità Militare, Roma, Italy; and †World Health Organization, Geneva, Switzerland

### Reference

1. Heymann DL, Rodier GG. Global surveillance of Communicable Diseases. Emerg Infect Dis 1998;4:362-5.

### Dual Infection with *Ehrlichia chaffeensis* and a Spotted Fever Group Rickettsia: A Case Report

**To the Editor:** In their article, Daniel J. Sexton et al. state, "Well- documented cases of simultaneous human infections with more than one tick-borne pathogen are rare" (1) and mention only two reports of such cases. However, another report should be mentioned because of its historical interest and the lessons it may teach.

In 1900 to 1905, in the Bitter Root Valley, a tick-borne disease emerged, which became known as Rocky Mountain spotted fever. Although Ricketts et al. later published a report (2), which identified the causative agent, in 1904 L.B. Chowning and W.M. Wilson published Studies on *Pyroplasma hominis* (3). They reported finding *Pyroplasma* (since changed to *Babesia)* in the blood of approximately 20 patients with spotted fever. They studied this organism in detail and even found the reservoir for it in the local rodent species. Wilson et al. thought that the organism was the causative agent of spotted fever. On the basis of their excellent plates and descriptions, it is clear that the organism they were describing was what we later came to know as *Babesia microti.*

The work of Wilson and Chowning was ignored and forgotten for many years. They had incorrectly concluded that spotted fever was caused by a parasite. For many years it was "well known" that *Babesia* infections became apparent in human patients only on removal or inactivation of the spleen. That persons with functional spleens were subject to infection with *B. microti* was finally established by the so-called Nantucket outbreak (4) and subsequent publications.

Therefore, Wilson and Chowning's work reports several cases of simultaneous infections of humans by two tickborne pathogens; i.e., patients had spotted fever and *B. microti* in the blood. More poignant was that an "emerging" disease of humans was missed and not discovered again for some 70 years.

**Alexander J. Sulzer**
Fellow, American Academy of Microbiology; Fellow, emeritus, Royal Society of Tropical Medicine and Hygiene; Member, emeritus, American Society of Tropical Medicine and Hygiene

### References

1. Sexton DJ, Corey GR, Carpenter C, Kong LQ, Gandhi T, Breitschwerdt E, et al. Dual infection with *Ehrlichia chaffeensis* and a spotted fever group rickettsia: a case report. Emerg Infect Dis 1998;4:311-6.
2. Ricketts HT. Some aspects of Rocky Mountain spotted fever. Rev Infect Dis 1909;1227-40.
3. Wilson LB, Chowning WM. Studies on *Pyroplasma hominis.* Rev Infect Dis 1904;1:31-57.
4. Ruebush TK, Juranek DD, Chisholm ES, Snow PC, Healy GR, Sulzer AJ. Human babesiosis on Nantucket Island. N Engl J Med 1977;297:825-87.

